# Situating Food Industry Influence: Governance Norms and Economic Order: Comment on "‘Part of the Solution’: Food Corporation Strategies for Regulatory Capture and Legitimacy"

**DOI:** 10.34172/ijhpm.2022.7197

**Published:** 2022-05-31

**Authors:** Raphael Lencucha

**Affiliations:** School of Physical and Occupational Therapy, Faculty of Medicine, McGill University, Montreal, QC, Canada.

**Keywords:** Commercial Determinants of Health, Food Corporations, Food Systems, Corporate Social Responsibility, Economic Policy, Health Governance

## Abstract

Lacy-Nichols and Williams provide important new insights into the ongoing contest over policy space and consumer behavior. I attempt to situate these insights in relation to government mandates and governance norms and situate these norms and mandates in the prevailing economic order. This approach is necessary to understand how corporate practices persist and why governments are receptive to the approaches outlined in the analysis conducted by Lacy-Nichols and Williams. This approach can help explain why governments are often receptive to corporations positioning themselves as ‘part of the solution’. Governments want strong economies and big food positions itself as contributor to this end. The point I attempt to articulate is that we often conceive of corporate power as *power over*, while I suggest that corporate power is rather *power within* and *through* a system that is oriented towards profits and economic growth.

## Introduction

The activities of tobacco, food, and alcohol corporations continue to perturb our sensibilities as public health scholars and advocates by promoting unhealthy products and undermining efforts to control these products. Lacy-Nichols and Williams introduce the adaptive approaches taken by ‘big food’ to position itself as part of the solution.^1^ The authors illustrate how food corporations have added to familiar strategies of direct and indirect opposition to regulation by building relationships with powerful actors and advancing new market strategies. The authors argue that unlike approaches that directly oppose or contest government regulation of their products and practices, this recent approach by corporations is characterized by “appeasement, co-option and partnership.”^1^ This new approach “serves to reinforce the industry’s economic and political power” (p. 847) while fostering an image of “‘good citizen’ willing to adapt for health goals” (p. 851).^1^ The authors further claim that this corporate approach has ultimately “fostered greater ambivalence from public health and government stakeholders” (p. 852) and has even served to “appease and pacify the public health community” (p. 851).^1^ This analysis sheds new light on the ongoing contest over policy space and consumer behavior, a contest that extends at least seven decades to the beginning of public health’s war against the tobacco industry.^2^ While the analysis of corporate influence over consumer and policy environments has gained a new name in the Commercial Determinants of Health (CDoH), the scholarship builds upon decades of systematic interrogation and exposure of the ways that corporate entities deliberately deceive publics when their products and practices are harmful to health, environment and social well-being, adapt their promotion efforts to ensure that they continue to attract customers, while at the same time strive to capture government regulatory space.^2,3^ This body of scholarship, like this analysis by Lacy-Nichols and Williams, often focuses attention squarely on the industry itself.

In this commentary I suggest that more attention needs to be paid to situating the existence and practices of corporations within the norms and systems that perpetuate their harmful products and practices. This situated approach can help explain how governments and other actors can come to view corporations and the strategies illustrated by Lacy-Nichols and Williams as legitimate, and illuminates the conditions that allow these approaches to persist. The challenge for CDoH researchers is to merge four approaches to corporate influence: a critical approach to corporate activity, a clear articulation of the systemic drivers or at least facilitators of this activity, an analysis of the common and often ineffective deference paid to corporations by governments (call it capture or giving the benefit of the doubt^4^), and finally creative and positive approaches to transforming systems.

## From Corporate Activity to Corporate Activity in Context

The ‘part of the solution’ approach of food corporations extends deeper than the strategies themselves and into norms that govern the relationship between market, state and society. As the authors note, corporate activity is tied to the “pursuit of profits and power” (p. 847). When industry opposes regulation, forms partnerships to “disarm criticism” (p. 847), or does all in its power to preserve, enhance, or salvage a positive public image we can confidently say that it is *doing what it is supposed to do*. The industry has a common playbook of strategies to oppose regulation, ingratiate publics and governments, and control the market. While the focus is largely on identifying the strategies themselves, it is also important to situate these strategies to better understand how and why they are often effective at advancing industry agendas.

An ongoing puzzle for CDoH scholars is to understand how industry can continue to overtly harm human health and the environment with seemingly unrelenting persistence and then, as Lacy-Nichols and Williams astutely illustrate, successfully position themselves as ‘part of the solution’. One question that rises from this analysis is why do governments seem so amenable to the approaches of food industry, even, as the authors note, supporting the pledges and self-regulatory gestures of corporations when these corporations largely continue producing and promoting the same unhealthy foods? One critical dimension to understand this situation is to look to the mandates of the economic sectors of government and the norms of governance. It is widely known that sectors within government, like trade and industry, support industry in different ways including by choosing not to regulate. The mandates of these sectors often explicitly require this support.^5^ The emphasis on economic development and growth absent any particular values of health and societal well-being remains a prominent feature of public policy in economic sectors.^6^ One example of this orientation by government sectors is the formal inclusion of corporate interests, including those known to cause harm to human health like the tobacco industry, in government decision-making spaces.^7,8^ If we see the inclusion of tobacco companies in such agencies, then it is less surprising to see food company representation given the relative preservation of a positive public image by these companies.

Industry maintains this position as ‘legitimate stakeholder’ in part by attempting to preserve its credibility as supplier of a desired product and as a contributor to wider economic goals, including employment or revenue generation. Lacy-Nichols and Williams expand widely known strategies to include strategies designed to position industry not only as innocent purveyor of products and economic contributor, but also as ‘part of the solution’ to the problems that stem from their own products and practices. While there is some recognition by some corporations that their products do in fact cause harm, a reluctant acceptance, there remains a more common deflection of responsibility from the industry back to consumer.^9,10^ The scholarship on obesogenic environments has countered industry attempts to shift responsibility to consumers by de-centering the individual and illustrating the power of environmental factors in shaping food choices and behaviors.^11^ Critical scholarship like that advanced by Herrick goes further to argue that the “risk of developing a chronic disease cannot be reduced to a set of (harmful) products and ‘product environments,’” emphasizing rather how industries actively perpetuate risk through their actions.^12^ This is an important point. The risk of allowing unhealthy commodity producing industries to shape the policy and consumer space is that they often distract attention from broader profit-seeking, health harming strategies, by drawing attention to individual products or product categories. For example, industry attempts to generate ‘lower risk’ products like low-sodium or low-fat food products or low-tar tobacco products, is one deceptive practice that has been shown to misdirect consumer attention away from the fact that the products often remain harmful. These attempts by industry to reformulate products is often coupled with promises of change that ultimately attempt to stall regulation in order to sustain profits. Food corporations have also used ‘enhanced nutrition’ strategies (ie, product reformulation, fortification, functionalization) to not only create a positive public image, but also to enter new markets.^13^ The rise of the Foundation for a Smoke-Free World funded in large part by Philip Morris International illustrates how corporations can successfully rebrand themselves as part of the solution when faced with unfriendly regulatory environments, even garnering the support of governments and tobacco control advocates. The example of the Foundation for a Smoke-Free world is an important one that illustrates that such efforts only thinly mask the core business; in this case generating profits from the sale of cigarettes.^14^

The challenge with this situation is that with profits comes power, through the ability to mobilize extensive resources to pursue these market and non-market strategies of influence.^15,16^ While we often conceive of corporate power as *power over, *when we situate corporate activity in the broader economic system that emphasizes continuous growth, often through capital accumulation and re-investment, we can reposition corporate power as *power within*. Efforts to become part of the solution are sensitized by mandates and norms of governance that already view corporations and beneficial to economies and societies.^17,18^ Food companies do particularly well at positioning themselves as part of the solution in part because food has become an ambiguous category. A company like Pepsi or Kraft can argue that they are providing staple products, products necessary for human sustenance, because of this broad notion of what is considered ‘food’. These companies attempt to contribute to school lunch programs or other ‘social goods’ based on the fact that ‘food’ is a necessity. At the same time the ‘social good’ is coupled with the argument that they contribute to the ‘economic good’ of countries. Food companies have become some of the largest companies in existence. In 2020, Pepsi Co was ranked fiftieth of all existing companies based on market value and employed almost 300 000 individuals.^19,20^ This is appealing to governments within a paradigm of crude economic growth or the belief that profits drive employment and investment. These companies leverage their ‘economic contribution’ as a way to legitimize pledges for self-regulation and reformulation, with the effect of “pacify(ing) public health pressure for regulatory or legislative action” (p. 848).^1^ Importantly, these corporations continue to generate the most revenue from their unhealthiest products. If we return to Pepsi Co as an example, we find that the North American segment of their beverage operations (ie, sugar-sweetened beverages) accounted for one-third of their total revenue of approximately 70 billion dollars in 2020. Non-alcoholic beverages like sugar-sweetened beverages are a massive income earner. The net profit margins (~15% in 2019) are some of the highest of any industry in the world.^21^

While corporate activity is a critical dimension to understanding consumer environments, the actions of corporations need to be situated within a system that places value on profits and employment, often over health and social protections. As Labonte notes, we need to widen our gaze to begin imagining how we can tame a capitalist system “that has outgrown its emancipatory promise and, in its most recent illiberal iteration, now threatens the very bases of human life.”^22^ Part of the challenge of the current economic order, one that feeds the legitimacy of ‘big food’ is the unqualified emphasis on growth. As Banerjee and colleagues note at the beginning of their special issue on a post-growth era, a narrow emphasis on growth (absent explicit emphasis onhealth, environmental protection, and social well-being) has been “canonized, often unwittingly, in everyday life” (p. 339) and scholarship.^23^ As Labonte further notes, “there are policy roadmaps for taming” this economic order “but only hints (circular economies, cooperatives) of its more thorough transformation.”^22^ It is the pervasiveness of this economic paradigm, one that shapes government mandates and governance norms, that makes it difficult to control the problematic practices of industry. Industry is partly viewed as part of the solution to the health problem because it is seen as a legitimate contributor to the economy.

## Conclusion

 Lacy-Nichols and Williams have brought forward important insights into how food corporations operate to maintain their core business when the potential for further regulation looms large. These insights are critically important if we are to continue to monitor and inform critiques of current food systems and continue to reimagine transformation. I have attempted to situate these insights in relation to government mandates and the prevailing norms of governance. This situated approach is necessary to understand how and why corporate practices persist and why governments are often receptive to these practices. If we are truly going to transform food systems towards healthier, more environmentally sustainable, and socially just processes and outcomes we need to incorporate the broader economic order and ways that governments are tied to this order in our approach to analysis and intervention.

## Ethical issues

 Not applicable.

## Competing interests

 Author declares that he has no competing interests.

## Author’s contribution

 RL is the single author of the paper.
